# Case Report: Anesthetic management of two dogs with third-degree atrioventricular block undergoing elective non-cardiac surgery under temporary transvenous pacing

**DOI:** 10.3389/fvets.2026.1856479

**Published:** 2026-05-29

**Authors:** Bernadette Vogel, Nicolai Hildebrandt, Kai Kneissl, Franz Söbbeler, Matthias Schneider

**Affiliations:** 1 Anesthesiology Department, Small Animal Clinic, Justus-Liebig-University Giessen, Giessen, Germany; 2 Cardiology Department, Small Animal Clinic, Justus-Liebig-University Giessen, Giessen, Germany; 3 Stomatological Department, Small Animal Clinic, Justus-Liebig-University Giessen, Giessen, Germany

**Keywords:** AV block, bradyarrhythmia, canine, pacemaker anesthesia, perioperative management

## Abstract

Anesthesizing patients with severe bradycardia caused by conduction disturbances poses a substantial perioperative risk and requires immediate access to pacing and advanced hemodynamic support. Two dogs were presented with a third-degree atrioventricular block for elective non-cardiac surgery. During anesthesia, temporary transvenous pacing was instituted to provide rate control and enable rapid intervention in response to perioperative hemodynamic changes. Both dogs remained clinically stable and recovered without major peri- or postoperative complications. The temporary pacing device was successfully removed once the dogs were fully awake, without incident. These two cases suggest that temporary transvenous pacing may be a feasible bridging strategy to facilitate safe anesthetic management in selected dogs with third-degree atrioventricular block, when performed by experienced personnel under continuous monitoring.

## Introduction

Third-degree atrioventricular (AV) block is a severe conduction disorder in dogs and is commonly associated with hemodynamic alterations that may result in weakness, syncope, or sudden cardiac death (1). Without intervention, the prognosis is poor, and permanent pacemaker implantation is considered the treatment of choice for patients with clinical signs who are unresponsive to medical treatment (2, 3). Patients with AV block are high-risk candidates for anesthesia, as severe anticholinergic-unresponsive bradycardia may reduce cardiac output despite a compensatory increase in stroke volume, compromising systemic and myocardial oxygen delivery (4).

In human medicine, temporary transvenous pacing (TTP) is a well-established strategy to stabilize patients with conduction disturbances during elective or emergency surgery (5). In contrast, in veterinary medicine, temporary pacemakers are usually employed in emergency situations or as a bridge to permanent pacemaker implantation (2). To the authors’ knowledge, their use to facilitate elective non-cardiac surgical procedures in dogs with severe conduction disorders has not been reported.

## Case description

### Case 1

A 7-year-old, 14-kg, male neutered mixed-breed dog was referred for an oral examination, dental scaling, and extraction of severely affected teeth due to severe periodontitis and stomatitis. The dog had initially presented to the referring veterinarian for decreased food intake and halitosis, without clinical signs suggestive of cardiac disease. Marked bradycardia prompted further cardiological assessment, including electrocardiography (ECG) and echocardiography. An ECG revealed a third-degree AV block with a ventricular escape rhythm at a rate of 45 beats per minute (bpm). Echocardiography showed moderate myxomatous mitral valve disease, classified as ACVIM stage B1, with normal left atrial and ventricular dimensions and a normalized left ventricular diastolic index (LVDd-n of 1.69; reference interval 1.27–1.85; LA/Ao 1.11; reference range < 1.60) (6, 7). A preanesthetic examination revealed a heart rate (HR) of 52 bpm, a grade II/VI systolic heart murmur over the mitral valve, a respiratory rate of 24 breaths per min, and a temperature of 38.6 °C. Pre-anesthetic bloodwork showed mildly increased alanine aminotransferase activity (116 U L^−1^; reference <100 U L^−1^) and an elevated pro-BNP concentration (1885 pmol L^−1^; > 1,500 pmol L^−1^ indicates high risk of developing heart failure within 12 months). C-reactive protein, leukocyte count, and fibrinogen concentration were within the reference intervals.

A peripheral intravenous catheter (22-G) was placed in the right cephalic vein, and the thorax was clipped to allow the placement of adult defibrillation/pacing pads while the dog was still conscious. After premedication with fentanyl (3 μg kg^−1^ IV), midazolam (0.5 mg kg^−1^), and atropine (0.04 mg kg^−1^ IV), the dog subsequently showed vocalization, paddling, and slight arousal. Alfaxalone (1.5 mg kg^−1^ IV, titrated to effect) was administered to achieve deep sedation. Ampicillin 25 mg kg^−1^ IV was administered prior to induction and was repeated perioperatively every 90 min. Oxygen supplementation was provided via a diaphragm-sealed face mask at a flow rate of 2 L min^−1^. During premedication, monitoring consisted of pulse oximetry with a display of the plethysmographic waveform. After adequate sedation, an ECVIM-CA cardiology Diplomate placed a 5F temporary transvenous pacemaker (TTPM; Abbott) via a 5F introducer sheath (Merit Medical), which was placed in the right jugular vein. Isolated ventricular premature complexes occurred during TTPM placement. Fluoroscopy confirmed appropriate right ventricular placement, and the lead was secured using a Roman sandal suture. The TTPM was programmed in VVI mode (ventricular pacing and sensing with inhibition in response to intrinsic ventricular activity) at 70 bpm, with a sensing amplitude of 3.5 mV and pacing output of 5 V, producing effective ventricular capture. The pacemaker placement and fixation took approximately 30 min. Apart from marked bradycardia (42 bpm) until pacing was established, no clinically relevant abnormalities were observed, and no intraoperative pacemaker adjustments were required.

Anesthesia was induced with alfaxalone (1 mg kg^−1^ IV) over 30 s, and orotracheal intubation was performed with an endotracheal tube (7.5 mm I. D. PVC). Anesthesia was maintained with sevoflurane in oxygen and air. An additional intravenous catheter was placed in the left cephalic vein (20-G), and invasive arterial blood pressure (IBP) monitoring was established using a 22-G catheter placed in the dorsal metatarsal artery. Monitoring included percutaneous ECG, IBP, pulse oximetry (SpO_2_), inspired oxygen fraction (FiO_2_), end-tidal inhalant concentration (EtAA), end-tidal carbon dioxide concentration (EtCO_2_), and rectal temperature. A crystalloid infusion was started at 5 mL kg^−1^ h^−1^. In addition to fentanyl (5 μg kg^−1^ h^−1^ IV), dobutamine (3 μg kg^−1^ min^−1^ IV) was administered as a constant rate infusion (CRI) to provide preemptive inotropic support and maintain cardiac output. Left maxillary, left inferior alveolar, and right mental nerve blocks were performed with mepivacaine 2% (total dose: 1 mg kg^−1^).

The palpebral reflex returned 40 min after induction, accompanied by an increase in mean arterial pressure (MAP) from 74 mmHg to 87 mmHg, interpreted as insufficient anesthetic depth in the context of surgical stimulation. EtAA was 0.9 vol% at that time. Alfaxalone (0.5 mg kg^−1^ IV) was administered, followed by volume-controlled ventilation (TV 14 mL kg^−1^, PIP 8–10 cmH₂O) because spontaneous ventilation did not resume. The respiratory rate was adjusted to maintain EtCO_2_ between 35 and 42 mmHg.

Dobutamine CRI was tapered to 1 μg kg^−1^ min^−1^ after MAP increased above 80 mmHg. Because the MAP remained stable above 75 mmHg, the dobutamine CRI was discontinued. Mechanical ventilation was discontinued 120 min after TTPM placement, when the dog showed signs of spontaneous breathing.

The stomatological procedure lasted 90 min, and the total anesthesia time was 130 min. At the end of anesthesia, sevoflurane was discontinued, and the patient was transferred to the recovery room. The pacemaker rate was gradually tapered from 70 to 50 bpm. The dog remained hemodynamically stable (MAP >80 mmHg, intrinsic HR 52 bpm) during recovery, and the pacemaker and arterial catheter were removed. Supportive warming was provided (rectal temperature 36.2 °C), and intravenous fluids were discontinued.

Post-anesthetic monitoring included ECG, non-invasive blood pressure (NIBP), and rectal temperature. The ECG confirmed a persistent third-degree AV block with a ventricular escape rhythm (48 bpm). The dog remained clinically stable and showed no signs of weakness or syncope. All other vital parameters were within physiological ranges. The dog was discharged 6 h after extubation with meloxicam 0.1 mg kg^−1^ PO q24h and amoxicillin-clavulanic acid (12.5 mg kg^−1^ PO q12h). An appointment for cardiological follow-up and the implantation of a permanent pacemaker was scheduled.

At the 30-day telephone follow-up, the owner reported an uneventful recovery with no clinical signs suggestive of worsening cardiac disease.

### Case 2

A 10-year-old, 15.2 kg, male, neutered mixed-breed dog with asymptomatic third-degree AV block and bradycardia (50 bpm) was referred for a partial mandibulectomy due to a right mandibular acanthomatous ameloblastoma.

Initial clinical examination revealed bradycardia at 48 bpm and a systolic heart murmur grade IV/VI with point of maximal intensity over the mitral valve. A cardiac examination revealed an atropine-unresponsive third-degree AV block with a ventricular escape rhythm, a moderate mitral and tricuspid regurgitation, normal left- and right-sided cardiac dimensions (LVDd-n 1.63; LA/Ao 1.03), and a tricuspid valve regurgitation velocity of 3.4 m/s, indicating a moderate probability of mild pulmonary hypertension according to ACVIM consensus guidelines (8). Bloodwork demonstrated a mild increase in globulin concentration (42 g L^−1^; reference <37.4 g L^−1^) and an elevated C-reactive protein (13.1 mg L^−1^; reference <12.2 mg L^−1^).

Prior to anesthesia, a 20-G peripheral intravenous catheter was placed in the left cephalic vein. Premedication consisted of midazolam (0.5 mg kg^−1^ IV) and fentanyl (5 μg kg^−1^ IV). Following premedication, alfaxalone (1 mg kg^−1^ IV) was administered to deepen sedation. Ampicillin (25 mg kg^−1^ IV) was administered and repeated every 90 min perioperatively. Oxygen supplementation was provided via a diaphragm-sealed face mask at 2 L min^−1^, and pulse oximetry with a plethysmographic waveform display was used for monitoring. A board-certified cardiologist (ECVIM-CA Cardiology) placed a 5F TTPM (Abbott) via a 5F introducer sheath (Merit Medical) in the right jugular vein. No arrhythmias occurred during placement. Fluoroscopy confirmed the right ventricular apical lead position, and the lead was secured as described for case 1. Placement and fixation were completed within approximately 25 min. The TTPM was programmed in VVI mode at 70 bpm, with a sensing amplitude of 4.0 mV and a pacing output of 3 V. During placement, apnea occurred, requiring endotracheal intubation with an endotracheal tube (7.5 mm I. D. PVC) and initiation of pressure-controlled ventilation (PIP 10 cmH_2_O). Ventilation was adjusted by modifying the pressure and respiratory rate to maintain an EtCO_2_ between 35 and 42 mmHg. Anesthesia was maintained with isoflurane in oxygen and air, along with fentanyl CRI at 5 μg kg^−1^ h^−1^. Hemodynamic support was initiated immediately after induction with a crystalloid infusion at 5 mL kg^−1^ h^−1^ and a dobutamine CRI starting at 1.5 μg kg^−1^ min^−1^. Monitoring included ECG, NIBP, SpO_2_, FiO_2_, EtAA, EtCO_2_, and rectal temperature.

After TTPM placement, a computed tomography (CT) scan was performed in sternal recumbency for surgical planning, followed by the placement of a second 20-G intravenous catheter in the right lateral saphenous vein, and a 22-G catheter was placed in the dorsal metatarsal artery for IBP monitoring.

Before surgery, dental scaling and the extraction of two teeth were performed in left and right lateral recumbency. Combining dental treatment and surgery in one anesthetic episode was intended to reduce bacterial contamination and avoid a second general anesthesia. During dental treatment, a decreased HR and broad QRS complexes were observed, suggesting a loss of capture. Effective pacing was restored by increasing the pacing output to 20 V. Therefore, repeated fluoroscopy was not performed. Five min after the loss of capture, the output was gradually decreased to 10 V, which maintained effective pacing. Loss of capture recurred when the output was reduced below 7.5 V.

Sixty min after induction, MAP gradually decreased, while EtAA was 1.0 vol%, prompting an increase in pacing rate from 70 to 90 bpm and a dobutamine CRI to 3 μg kg^−1^ min^−1^. Because the MAP remained <65 mmHg, a norepinephrine CRI was initiated at 0.05 μg kg^−1^ min^−1^ and titrated to maintain a MAP >65 mmHg. After completion of the dental treatment, an ultrasound-guided trigeminal nerve block with 0.4 mL of ropivacaine 0.5% was performed.

Surgery for hemimandibulectomy started 145 min after induction. At 180 min, the MAP increased rapidly to 100 mmHg, suggesting insufficient trigeminal block efficacy. Fentanyl (5 μg kg^−1^ IV) was administered, the fentanyl CRI was increased to 10 μg kg^−1^ h^−1^, and norepinephrine was discontinued. As the MAP rose to 110 mmHg, ketamine (1 mg kg^−1^ IV) was administered, followed by a ketamine CRI at 10 μg kg^−1^ min^−1^, which restored hemodynamic stability (MAP 65–85 mmHg). At the end of surgery, the fentanyl and ketamine infusions were reduced to 5 μg kg^−1^ h^−1^ and 5 μg kg^−1^ min^-1,^ respectively, and isoflurane was discontinued, marking a total anesthesia time of 210 min. The pacemaker rate was lowered from 90 to 75 bpm, and a nasogastric feeding tube was placed.

During recovery, the pacemaker rate was gradually reduced to 50 bpm over 15 min, which was well tolerated hemodynamically. Once the patient regained consciousness, the TTPM was removed, and the intrinsic HR remained between 45 and 50 bpm. During the first 24 h after anesthesia, continuous ECG monitoring was performed, and respiratory rate, mucous membrane color, capillary refill time, rectal temperature, and NIBP were assessed every 2 h. Crystalloid infusion was reduced to 2 mL kg^−1^ h^−1^. Post-operative analgesia consisted of a fentanyl CRI (2.5 μg kg^−1^ h^−1^), a ketamine CRI (5 μg kg^−1^ min^−1^), and pregabalin (4 mg kg^−1^ PO q8h). Meloxicam (0.1 mg kg^−1^ PO q24h) was added to the analgesic plan 1 day after surgery.

The dog recovered uneventfully during hospitalization. A cardiological recheck prior to discharge revealed no evidence of worsening myocardial function. The patient was discharged 2 days after surgery with a prescription for amoxicillin-clavulanic acid, meloxicam, and pregabalin.

At the 30-day recheck, the owner reported an uneventful recovery after anesthesia without clinical signs of the underlying condition.

## Discussion

The present cases highlight a clinically relevant scenario in which elective, non-cardiac surgery was performed on dogs with untreated third-degree AV block using TTPM as a peri-anesthetic bridging strategy. Elective surgery may be necessary for these patients, but anesthesia poses a high risk because the ventricular escape rhythm is often fixed and unable to compensate for anesthetic-induced cardiovascular changes (9, 10).

In both patients, primary or secondary sinus or AV nodal dysfunction was suspected rather than vagally mediated AV block (11). In case 2, atropine testing further reduced the likelihood of a vagally mediated cause. Therefore, atropine was only used in case 1, where atropine responsiveness was unclear.

Published data on complications of TTP in veterinary medicine are scarce. Reported human complications include loss of capture due to lead dislodgement, ventricular arrhythmias, infection, thrombosis, and myocardial perforation during lead placement (12, 13). In both dogs, the pacemaker lead was placed aseptically by a board-certified cardiologist, antimicrobial prophylaxis was administered, and no clinical signs of jugular vein thrombosis or endocarditis occurred, although follow-up for jugular vein thrombosis was not performed. In the second case, transient loss of capture was observed, possibly promoted by repeated changes in recumbency. Because a passive lead was used, loss of capture due to lead tip movement was possible and suspected in this case, whereas other causes reported in permanent pacemaker patients, including local inflammation, fibrosis, neoplasia, lead fracture, or dislodgement, are less common with temporary pacing (14, 15).

Several pacing methods have been described in dogs, including transvenous, transcutaneous, and transoesophageal pacing; the latter is mainly used for patients with intact AV conduction (11). Transvenous pacing was chosen over transcutaneous pacing because it provides more reliable ventricular capture, greater patient comfort, and precise programmability over prolonged periods (16, 17). However, transcutaneous pacing remains a valuable option as a rapid, non-invasive bridge in emergency cases (18). In both cases, VVI pacing provided reliable rate support by stimulating and sensing the ventricle and inhibiting output after sensing spontaneous ventricular depolarization (19). Although a lack of atrial sensing may reduce cardiac output through atrioventricular asynchrony and loss of the atrial kick, the MAP remained clinically acceptable, and hypotension responded to titrated catecholamines and anesthetic-depth adjustments (20).

In both cases, midazolam and fentanyl as premedication provided anxiolysis, sedation, and analgesia with limited cardiovascular depression and available antagonists. Benzodiazepines have minimal cardiovascular effects at clinical doses; however, higher doses (e.g., 1 mg kg^−1^) may mildly increase HR and reduce blood pressure (21, 22). Paradoxical excitation may occur when sedation is inadequate and may have contributed to vocalization and paddling in case 1, although atropine administration precluded clear attribution (23, 24). Fentanyl was chosen for its potent analgesia, short duration, CRI titratability, and MAC-sparing effect, although it may also increase vagal tone and promote bradyarrhythmias (25). Alternative premedication strategies could have included fentanyl alone or the omission of midazolam, with alfaxalone titrated once monitoring was established.

General anesthesia was induced with slow intravenous alfaxalone titration to effect, allowing careful adjustment of anesthetic depth in patients with potentially delayed drug distribution and clearance due to low cardiac output (26). The relatively high dose in case 1 likely reflected paradoxical excitation and temperament despite the preceding premedication. Although the current evidence does not show the superiority of alfaxalone over other hypnotics in dogs with third-degree AV block, alfaxalone was preferred over propofol because it could be easily titratable and was considered to provide acceptable cardiovascular stability when administered to effect (27, 28). Etomidate is a valuable choice due to its minimal effects on myocardial contractility and systemic vascular resistance, but it was unavailable and not routinely used by the team (29). In case 1, additional alfaxalone was given during maintenance when the anesthetic depth was considered insufficient. Fentanyl titration may have been an alternative with less cardiovascular depression.

Anesthesia was maintained with volatile agents despite their dose-dependent cardiovascular depression because inhalant anesthesia was familiar, readily titratable, and practical during procedures with variable duration and stimulation (30, 31). However, total intravenous anesthesia may have offered cardiovascular advantages; therefore, both cases were managed with a balanced approach to reduce inhalant requirements (32). Fentanyl CRI was used because of its potent, titratable, reversible, and inhalant-sparing analgesic effects (33). Its potential to increase vagal tone was considered acceptable because ventricular rate support was provided by TTPM. Ketamine and lidocaine were not used as first-line adjuncts because ketamine may increase sympathetic tone and myocardial oxygen demand, whereas lidocaine may affect ventricular conduction (34, 35). Ketamine was added in case 2 when inadequate analgesia was suspected despite the use of fentanyl and locoregional anesthesia.

Local anesthetic blocks were used to provide surgical-site desensitization and reduce the need for inhalants (36). In case 2, a trigeminal nerve block was selected because the caudal extent of the mandibular resection was determined intraoperatively, and broader desensitization was desired (37). However, the limited evidence supporting this technique and the need for higher fentanyl doses and adjunctive ketamine suggested incomplete analgesia. In retrospect, an additional mandibular nerve block could have been performed intraoperatively.

Low-dose dobutamine was administered during and after anesthesia induction, before hypotension occurred, to support myocardial contractility and cardiac output via *β*_1_-adrenergic agonism and to mitigate anesthetic-induced negative inotropy (38, 39). Although evidence for prophylactic inotrope use is limited, early inotropic support may be justified for high-risk cardiac patients, as rapid hemodynamic deterioration in this population may be difficult to reverse. At the same time, the risk of β-adrenergic tachyarrhythmias and ventricular ectopy must be recognized (40–42). Therefore, small dose adjustments and continuous ECG monitoring are essential. At the doses used, improved cardiac output without an increased risk of ventricular arrhythmias has been reported (40).

However, when vasodilation predominates, dobutamine alone may not adequately restore MAP (43, 44). In case 2, norepinephrine was added because IBP showed persistently low diastolic arterial pressure, suggesting vasodilation. Norepinephrine counteracts inhalant-induced vasodilation via *α*_1_-mediated vasoconstriction and may support coronary perfusion by increasing diastolic arterial pressure (43). However, norepinephrine and dobutamine may increase myocardial oxygen demand, creating a potential mismatch between myocardial oxygen delivery and demand (45, 46). Therefore, cardiovascular support should be titrated to the lowest effective dose and tapered as soon as feasible.

## Conclusion

These cases demonstrate that TTPM, combined with careful drug selection, proactive hemodynamic support, and a multidisciplinary approach, can enable safe anesthesia for elective non-cardiac surgery in dogs with third-degree AV block. Although complications such as lead dislodgement must be anticipated, temporary pacing can provide effective peri-anesthetic rate support in selected high-risk patients.

## Data Availability

The original contributions presented in the study are included in the article/supplementary material, further inquiries can be directed to the corresponding author.
